# Virtual Funerals During COVID-19 and Beyond

**DOI:** 10.1093/geroni/igaa057.3530

**Published:** 2020-12-16

**Authors:** Ivy Muturi, Shannon Freeman, Davina Banner-Lukaris

**Affiliations:** University of Northern British Columbia, Prince George, British Columbia, Canada

## Abstract

Physical distancing measures and the restrictions on large group gatherings following the COVID-19 pandemic have left many to consider alternative approaches to commemorating the death of a loved one. Advancements in information technologies and the availability of affordable electronic devices have brought forth the ability to use virtual platforms to host funeral services for those unable to be with their loved ones. The aim of this study was to identify existing and potential online platforms for hosting a virtual funeral, explore the safety considerations of hosting a service during the pandemic and share the experiences of individuals who have previously hosted a virtual service. To conduct the research, an environmental scan was undertaken searching academic, grey literature and online websites. The results showed that there are currently several online platforms made specifically for virtual services and many free public platforms that can be used. Death services must ensure staff who are in direct contact with the deceased have proper Personal Protective Equipment and companies must adhere to regulations regarding group gatherings, screening for symptoms and physical distancing. Some individuals expressed having a positive experience, stating that the virtual service felt more intimate, while others expressed difficulty in navigating the technology, particularly the older adult attendees. Virtual funeral services may prove to be a practical and safer alternative during COVID-19 and may provide some comfort for those facing such challenging times. By examining existing platforms and their use, an opportunity exists to generate recommendations for additional supports, particularly for older adults.

